# Supplementary material for: Sex trafficking vulnerabilities in context: An analysis of 1,264 case files of adult survivors of commercial sexual exploitation
Source: PLoS One. 2024 Nov 20;19(11):e0311131. doi: 10.1371/journal.pone.0311131 (PMC11578464; doi:10.1371/journal.pone.0311131)
Supplement: S1 File — (DOCX) [file pone.0311131.s001.docx]

**Supplemental 1**

***Supplemental Literature Review***

**Theory**

In the case of CSE, feminist theory is fiercely divided into two factions: Neo-abolitionists and sex positivists.^1-2^ Sex positivists, or those who are pro-"sex work," criticize neo-abolitionists, stating that women should maintain full agency over their decisions about intimacy, their bodies, and the type of "work" they choose to pursue.^2^ On the other hand, neo-abolitionists view all forms of commercial sex as a form of violence against women and believe that sexual commerce can never be fully consensual.^3-4^ Specifically, radical feminism is a neo-abolitionist perspective that attributes the subordination and violence against women to patriarchy and male privilege.^1, 5^ Marxist feminism is another neo-abolitionist perspective that focuses on capitalism and the appropriation of women's sexual energy for profit.^6^ The political economy perspective is similar to Marxist feminism and acknowledges the impact of a society's economy on women living in poverty, requiring women to become more dependent on welfare, sexual partners, children's fathers, and other social supports.^7-8^ Such women may become vulnerable to abuse and exploitation.^4, 9-14^ These neo-abolitionist theoretical perspectives acknowledge the role of context on outcomes.

Because of the challenges associated with macro-level research, Weitzer^15^ encourages future researchers to study sexual exploitation from the micro, or individual-level perspective. Micro-level theories of CSE are generally descriptive and center around entry into and exit out of exploitation.^16^ Exit theories will not be discussed in this article as the nature of the secondary dataset does not allow for us to consider intentions related to exit. Previously considered entry theories include general strain theory,^17-18^ social control theory,^19-20^ social learning theory,^21-22^ lifestyle-exposure theory,^22-24^ and routine-activity theory.^22, 24-25^ These theories frame exploitation as deviant behavior rather than victimization and have only been discussed in relation to CSE in one publication.^16^ Instead, human behavior theory that considers agency embedded within context is necessary to fully understand vulnerability.

**Multisystem Frameworks**

**Ecological Model.** Bronfenbrenner's^26^ ecological theory is a theoretical framework that explains the ways in which individual, relational (i.e., familial or microsystem), social (i.e., community or exosystem), and societal (i.e., cultural experiences or macrosystem) factors shape the individual within it. Individuals are impacted by their own personal attributes, like biology, mental health, educational achievement, and past experiences. The relational level considers the immediate surroundings of an individual and includes events, developmental processes, and family relationships. Push and pull factors related to CSE at this level might include childhood sexual abuse, number of children, and low educational achievement. The community level considers the way in which the microsystem interconnects the individual and family to events in the broader institutions which structure society, such as the economic, educational, legal, mass media, military, and political systems. Number of arrests could be a factor that creates vulnerability for sexual exploitation at this level. Educational achievement may also be considered at this level if the reason for low educational achievement was low quality education in the individual's community. The societal level acknowledges the ideologies, organization, values, attitudes, and ways of thinking evident in a culture, which may vary by subculture within the society (e.g., social classes, ethnic, and religious groups). Feminist theory becomes relevant at this level. Though the macro level-factors are not represented in this present study, it is acknowledged that the idea of agency exists within all of these contexts. An additional consideration is the "Chronosystem," or the layer of time, in which an individual’s age and the ordering of events, context, and historical sequence identify the impact of prior experiences upon subsequent development. Age of entry into CSE and length of CSE could be relevant variables at this level.

**Life Course Theory.** Survivors of CSE experience these push and pull factors associated with exploitation that cycle throughout their lives. As with the ecological model, life course theory considers how time, culture, context, and the interdependence of family relationships influence people’s lives.^27^ Life course theory specifically acknowledges that agency is embedded in relationships, historical time and place, and events and that these contexts are not static, but evolve throughout a person's life.^28^ Life Course Theory has been suggested as a potential pathway from childhood to exploitation.^29-31^ Transitions and turning points typically involve a change in identity as well as circumstances that occur at both the individual and the family level. Arrest or taking on the role of mother are examples of these transitions and turning points that may create push and pull factors related to exploitation.

**Revictimization Theory**

Numerous studies have suggested that individuals who have experienced sexual abuse as children may be more likely to experience sexual abuse later in life.^32-33^ Franchino-Olsen^31^ proposes revictimization theory as a potential pathway connecting childhood sexual abuse and exploitation. Learned behavior, substance abuse, volatile interpersonal relationships, and socioeconomic statuses have been suggested as reasons for revictimization. However, Messman-Moore and Long^33^ recommend an ecological framework to acknowledge factors beyond the individual level, especially since individual agency is violated at the moment of abuse.

**Mental Health Diagnoses and CSE**

**Bipolar Disorder.** Bipolar disorder describes pervasive cycling between manic and depressive episodes that may be characterized by psychosis.^34^ It is estimated that approximately 4.4% of adults in the United States live with bipolar disorder.^35^ In a sample of minor victims of CSE, Palines et al. found that the rate of bipolar disorder was 26.6%, which was significantly higher (*p* < .001) than the rate of bipolar disorder for other at-risk groups (i.e., runaways, juvenile offenders, and children in foster care).^36^

**Neurodevelopmental Disorders.** The fifth edition of the *Diagnostic and Statistical Manual of Mental Disorders* (DSM) describes neurodevelopmental disorders, previously termed "learning disabilities," as conditions that may interfere with academic, occupational, personal, and social functioning that form before a child enters elementary school.^34^ Relevant neurodevelopmental disorders related to this study include attention deficit hyperactivity disorder (ADHD), autism spectrum disorder, and Dyslexia. The CDC estimates that the rate of ADHD in girls in the United States to be 5.6%.^37^ In their sample of male and female minor victims of CSE, Palines et al. discovered a rate of 52.4% for ADHD, which was also significantly higher (*p* < .001) than the rate of ADHD for other at-risk groups (i.e., runaways, juvenile offenders, and children in foster care).^36^ According to Autism Speaks, 1 in 116 girls is identified as having autism spectrum disorder.^38^ Though there are cases of individuals diagnosed with autism spectrum disorder who have experienced CSE, there are not enough data available to determine its rate among survivors.^39^ The rate of dyslexia in the United States is estimated to be between 3% and 7%.^40^ There are no data on the rate of dyslexia for survivors of CSE.

**Schizophrenia Spectrum Disorders.** Schizophrenia spectrum disorders and other psychotic disorders are characterized by abnormal body movements, delusions, disorganized thinking and speech, and hallucinations.^34^ Onset for schizophrenia can occur during one's late teens up until their early 30's. The National Institute of Mental Health estimates that the rate of schizophrenia in the United States is between 0.25% and 0.64%.^41^ Palines et al.'s sample of minor victims or CSE had a rate of 14% for psychosis.^36^

**S1 File References**

1. Dines G. The white man's burden: Gonzo pornography and the construction of black masculinity. Yale JL & Feminism. 2006;18:283.
2. Ferguson, A., Philipson, I., Diamond, I., Quimby, L., Vance, C. S., & Snitow, A. B. The feminist sexuality debates. Signs: Journal of Women in Culture and Society. 1984;10(1):106-25.
3. Tiefenbrun S. The saga of Susannah-A US remedy for sex trafficking in women: The Victims of Trafficking and Violence Protection Act of 2000. Utah L. Rev.. 2002:107.
4. Wilson B, Butler LD. Running a gauntlet: A review of victimization and violence in the pre-entry, post-entry, and peri-/post-exit periods of commercial sexual exploitation. Psychological trauma: theory, research, practice, and policy. 2014 Sep;6(5):494.
5. Farley M. Prostitution harms women even if indoors: Reply to Weitzer. Violence against women. 2005 Jul;11(7):950-64.
6. Miriam K. Stopping the traffic in women: Power, agency and abolition in feminist debates over sex‐trafficking. Journal of social philosophy. 2005 Mar;36(1):1-7.
7. Adelman M. The battering state: Towards a political economy of domestic violence. Journal of Poverty. 2004 Jul 22;8(3):45-64.
8. Edin K, Lein L. Making ends meet: How single mothers survive welfare and low-wage work. Russell Sage Foundation; 1997 Apr 17.
9. Anthias F. The intersections of class, gender, sexuality and ‘race’: The political economy of gendered violence. International Journal of Politics, Culture, and Society. 2014 Jun;27:153-71.
10. Farley M, Barkan H. Prostitution, violence, and posttraumatic stress disorder. Women & health. 2008 Aug 1;27(3):37-49.
11. Miller CL, Fielden SJ, Tyndall MW, Zhang R, Gibson K, Shannon K. Individual and structural vulnerability among female youth who exchange sex for survival. Journal of Adolescent Health. 2011 Jul 1;49(1):36-41.
12. Valera RJ, Sawyer RG, Schiraldi GR. Perceived health needs of inner-city street prostitutes: A preliminary study. American journal of health behavior. 2001 Jan 1;25(1):50-9.
13. Van Leeuwen JM, Hopfer C, Hooks S, White R, Petersen J, Pirkopf J. A snapshot of substance abuse among homeless and runaway youth in Denver, Colorado. Journal of Community Health. 2004 Jun;29(3):217-29.
14. Watson J. Understanding survival sex: Young women, homelessness and intimate relationships. Journal of youth studies. 2011 Sep 1;14(6):639-55.
15. Weitzer R. New directions in research on human trafficking. The ANNALS of the American Academy of Political and Social Science. 2014 May;653(1):6-24.
16. Gerassi L. A heated debate: Theoretical perspectives of sexual exploitation and sex work. Journal of sociology and social welfare. 2015 Dec;42(4):79.
17. Reid JA. Exploratory review of route-specific, gendered, and age-graded dynamics of exploitation: Applying life course theory to victimization in sex trafficking in North America. Aggression and Violent Behavior. 2012 May 1;17(3):257-71.
18. Agnew R. Foundation for a general strain theory of crime and delinquency. Criminology. 1992 Feb;30(1):47-88.
19. Gwadz MV, Gostnell K, Smolenski C, Willis B, Nish D, Nolan TC, Tharaken M, Ritchie AS. The initiation of homeless youth into the street economy. Journal of adolescence. 2009 Apr 1;32(2):357-77.
20. Hirschi T. Causes of Delinquency Univerisity of California Press. Berkeley, CA. 1969.
21. Patterson GR. Coercive family process, Castalia. Eugene, OR. 1982.
22. Whitbeck LB, Simons RL. A comparison of adaptive strategies and patterns of victimization among homeless adolescents and adults. violence and victims. 1993 Jan 1;8(2):135-52.
23. Hindelang MJ, Gottfredson MR, Garofalo J. Victims of personal crime: An empirical foundation for a theory of personal victimization. Cambridge, MA: Ballinger; 1978.
24. Tyler KA, Whitbeck LB, Hoyt DR, Cauce AM. Risk factors for sexual victimization among male and female homeless and runaway youth. Journal of Interpersonal violence. 2004 May;19(5):503-20.
25. Cohen LE, Felson M. Social change and crime rate trends: A routine activity approach (1979). InClassics in environmental criminology 2010 May 25 (pp. 203-232). Routledge.
26. Bronfenbrenner U. The ecology of human development: Experiments by nature and design. Harvard university press; 1979.
27. Allen KR, Henderson AC. Family theories: Foundations and applications. John Wiley & Sons; 2016 Sep 8.
28. Elder GH, Johnson MK, Crosnoe R. The emergence and development of life course theory. Springer US; 2003.
29. Cobbina JE, Oselin SS. It’s not only for the money: An analysis of adolescent versus adult entry into street prostitution. Sociological Inquiry. 2011 Aug;81(3):310-32.
30. Fedina L, Williamson C, Perdue T. Risk factors for domestic child sex trafficking in the United States. Journal of interpersonal violence. 2019 Jul;34(13):2653-73.
31. Franchino-Olsen H. Vulnerabilities relevant for commercial sexual exploitation of children/domestic minor sex trafficking: A systematic review of risk factors. Trauma, Violence, & Abuse. 2021 Jan;22(1):99-111.
32. Messman TL, Long PJ. Child sexual abuse and its relationship to revictimization in adult women: A review. Clinical Psychology Review. 1996 Jan 1;16(5):397-420.
33. Messman-Moore TL, Long PJ. The role of childhood sexual abuse sequelae in the sexual revictimization of women: An empirical review and theoretical reformulation. Clinical psychology review. 2003 Jul 1;23(4):537-71.
34. American Psychiatric Association [APA]. First MB. DSM-5® handbook of differential diagnosis. American Psychiatric Pub; 2013 Nov 19.
35. National Institute of Mental Health. Bipolar disorder [Internet]. 2022. Available from: https://www.nimh.nih.gov/health/statistics/bipolar-disorder#:~:text=Prevalence%20of%20Bipolar%20Disorder%20Among%20Adults,-Based%20on%20diagnostic&text=An%20estimated%204.4%25%20of%20U.S.,some%20time%20in%20their%20lives.
36. Palines PA, Rabbitt AL, Pan AY, Nugent ML, Ehrman WG. Comparing mental health disorders among sex trafficked children and three groups of youth at high-risk for trafficking: a dual retrospective cohort and scoping review. Child Abuse & Neglect. 2020 Feb 1;100:104196.
37. Danielson ML, Bitsko RH, Ghandour RM, Holbrook JR, Kogan MD, Blumberg SJ. Prevalence of parent-reported ADHD diagnosis and associated treatment among US children and adolescents, 2016. Journal of Clinical Child & Adolescent Psychology. 2018 Mar 4;47(2):199-212.
38. Autism statistics and facts [Internet]. [cited 2023]. Available from: https://www.autismspeaks.org/autism-statistics-asd#:~:text=Autism%20Prevalence,)%2C%20according%20to%202018%20data.&text=Boys%20are%20four%20times%20more,diagnosed%20with%20autism%20than%20girls.
39. Palermo MT, Bogaerts S. Sex selling and autism spectrum disorder: Impaired capacity, free enterprise, or sexual victimization? Journal of Forensic Psychology Practice. 2015 Aug 8;15(4):363-82.
40. Fletcher JM, Lyon GR, Fuchs LS, Barnes MA. Learning disabilities: From identification to intervention. Guilford Publications; 2018 Nov 21.
41. National Institute of Mental Health. Schizophrenia [Internet]. 2022. Available from: https://www.nimh.nih.gov/health/statistics/schizophrenia#:~:text=Across%20studies%20that%20use%20household,between%200.25%25%20and%200.64%25.
