# Supplementary material for: Sex trafficking vulnerabilities in context: An analysis of 1,264 case files of adult survivors of commercial sexual exploitation
Source: PLoS One. 2024 Nov 20;19(11):e0311131. doi: 10.1371/journal.pone.0311131 (PMC11578464; doi:10.1371/journal.pone.0311131)
Supplement: S1 Table — (DOCX) [file pone.0311131.s002.docx]

**S1 Table.**

***Mean Comparisons with U.S. General Population, Survivors of Commercial Sexual Exploitation (CSE), and the Present Study* (Table 3)**

|  | **General U.S. population** | **Survivors of**  **CSE** | **The present study** |
| --- | --- | --- | --- |
| Ever experience childhood sexual abuse | .250^1^ | .850^12^ | .710 |
| Age of entry | - | 18 - 20^13-15^ | 22.070 |
| Anxiety | .311^2^ | .009 - .196^16-17^ | .330 |
| Bipolar disorder | .044^3^ | .266^16^ | .380 |
| Number of children | 1.600^4^ | - | 1.880 |
| Cycling | - | 2.580^18^ | 1.277 |
| Depression | .084^5^ | .273 - .455^16-17^ | .420 |
| Educational Achievement |  |  |  |
| Less than high school diploma or equivalent | .089^6^ | .269^19^ | .381 |
| High school diploma or equivalent | .279^6^ | .495^19^ | .324 |
| Some college | .149^6^ | .237^19^ | .196 |
| Associates degree | .105^6^ |  |  |
| Bachelor's degree | .235^6^ |  | .041 |
| Advanced degree | .144^6^ |  |  |
| Length of exploitation | - | 8.700^20^ | 11.730 |
| Neurodevelopmental disorders | - | - | .110 |
| ADHD | .056^7^ | .524^16^ | .084 |
| Autism spectrum disorder | .009^8^ | - | .009 |
| Dyslexia | .03 - .07^9^ | - | .013 |
| Unspecified learning or cognitive disability | - | - | .015 |
| PTSD | .068^10^ | .890^21^ | .340 |
| Schizophrenia spectrum disorder | .003 - .006^11^ | .140^16^ | .130 |
| Trafficking | - | .840^22-23^ | .750 |

**S1 Table References**

1. Felitti VJ, Anda RF, Nordenberg D, Williamson DF, Spitz AM, Edwards V, Marks JS. Relationship of childhood abuse and household dysfunction to many of the leading causes of death in adults: The Adverse Childhood Experiences (ACE) Study. American journal of preventive medicine. 1998 May 1;14(4):245-58.
2. National Institute of Mental Health. Any anxiety disorder [Internet]*.* 2022a. Available from: https://www.nimh.nih.gov/health/statistics/any-anxiety-disorder#:~:text=Prevalence%20of%20Any%20Anxiety%20Disorder%20Among%20Adults,-Based%20on%20diagnostic&text=An%20estimated%2019.1%25%20of%20U.S.,than%20for%20males%20(14.3%25).
3. National Institute of Mental Health. Bipolar disorder [Internet]. 2022. Available from: https://www.nimh.nih.gov/health/statistics/bipolar-disorder#:~:text=Prevalence%20of%20Bipolar%20Disorder%20Among%20Adults,-Based%20on%20diagnostic&text=An%20estimated%204.4%25%20of%20U.S.,some%20time%20in%20their%20lives.
4. World Bank. Fertility rate, total (per woman) - United States [Internet]. 2020. Available from: https://data.worldbank.org/indicator/SP.DYN.TFRT.IN?locations=US
5. National Institute of Mental Health. Major depression [Internet]. 2022c. Available from: https://www.nimh.nih.gov/health/statistics/major-depressionNational Institute of Mental Health. (2022). *Mental health.* https://www.nimh.nih.gov/health/statistics/mental-illness#:~:text=Nearly%20one%20in%20five%20U.S.,mild%20to%20moderate%20to%20severe.
6. United States Census Bureau. Census bureau releases new educational attainment data [Internet]. 2022. Available from: https://www.census.gov/newsroom/press-releases/2022/educational-attainment.html#:~:text=In%202021%2C%2029.4%25%20of%20men,women%20and%2046.9%25%20were%20men.
7. Danielson ML, Bitsko RH, Ghandour RM, Holbrook JR, Kogan MD, Blumberg SJ. Prevalence of parent-reported ADHD diagnosis and associated treatment among US children and adolescents, 2016. Journal of Clinical Child & Adolescent Psychology. 2018 Mar 4;47(2):199-212.
8. Autism statistics and facts [Internet]. [cited 2023]. Available from: https://www.autismspeaks.org/autism-statistics-asd#:~:text=Autism%20Prevalence,)%2C%20according%20to%202018%20data.&text=Boys%20are%20four%20times%20more,diagnosed%20with%20autism%20than%20girls.
9. Fletcher JM, Lyon GR, Fuchs LS, Barnes MA. Learning disabilities: From identification to intervention. Guilford Publications; 2018 Nov 21.
10. Gradus JL. Epidemiology of PTSD from the National Center for Post-Traumatic Stress Disorder, Department of Veterans Affairs. From the National Center for Post-Traumatic Stress Disorder, Department of Veterans Affairs. Available online: www.ptsd.va.gov. 2013.
11. National Institute of Mental Health. Schizophrenia [Internet]. 2022. Available from: https://www.nimh.nih.gov/health/statistics/schizophrenia#:~:text=Across%20studies%20that%20use%20household,between%200.25%25%20and%200.64%25.
12. Farley M, Cotton A, Lynne J, Zumbeck S, Spiwak F, Reyes ME, Alvarez D, Sezgin U. Prostitution and trafficking in nine countries: An update on violence and posttraumatic stress disorder. Journal of trauma practice. 2004 Jan 14;2(3-4):33-74.
13. The Counter Trafficking Data Collaborative [CTDC]. Global data hub on human trafficking [Internet]. 2022. Available from: https://www.ctdatacollaborative.org/
14. May T, Harocopos A, Hough JM, Willis CF. For love or money: pimps and the management of sex work. London: Home Office, Policing and Reducing Crime Unit, Research, Development and Statistics Directorate; 2000.
15. Norton-Hawk MA. The counterproductivity of incarcerating female street prostitutes. Deviant Behavior. 2001 Sep 30;22(5):403-17.
16. Palines PA, Rabbitt AL, Pan AY, Nugent ML, Ehrman WG. Comparing mental health disorders among sex trafficked children and three groups of youth at high-risk for trafficking: a dual retrospective cohort and scoping review. Child Abuse & Neglect. 2020 Feb 1;100:104196.
17. Twill SE, Green DM, Traylor A. A descriptive study on sexually exploited children in residential treatment. In Child & Youth Care Forum 2010 Jun (Vol. 39, pp. 187-199). Springer US.
18. Furlong C, Rhulmann L. The association between poly-trauma victimization, perceptions of emotional support, and cycling for survivors of sex trafficking [Unpublished manuscript]. Department of Human Development and Family Science, Auburn University; 2019.
19. Cronley C, Cimino AN, Hohn K, Davis J, Madden E. Entering prostitution in adolescence: History of youth homelessness predicts earlier entry. Journal of aggression, maltreatment & trauma. 2016 Oct 20;25(9):893-908.
20. Kramer LA. Emotional experiences of performing prostitution. Journal of Trauma Practice. 2004 Jan 14;2(3-4):186-97.
21. Krumrei EJ, Fitzgerald K. The psychological correlates of engaging in prostitution. In Poster presented at the meeting of the American Psychological Association, Honolulu, HI; 2013.
22. Trafficking Victims Protection Act of 2000, 22 U.S.C. §§ 7101–7113 (2000).
23. Farley M, Franzblau K, Kennedy MA. Online prostitution and trafficking. Alb. L. Rev.. 2013;77:1039.
